# Supplementary material for: The potential of digital behavioural tests as a diagnostic aid for psychosis
Source: PLOS Digit Health. 2023 Sep 15;2(9):e0000339. doi: 10.1371/journal.pdig.0000339 (PMC10503702; doi:10.1371/journal.pdig.0000339)
Supplement: Attachment — Submitted filename: rebuttal_letter.docx [file pdig.0000339.s001.docx]

We would like to thank the editor and both reviewers for encouraging comments that helped us to improve the manuscript.

Specifically, comment on the (lack of) generalisability of the findings and the model choice.

Our assumption of the generalisability of the study from a sample to the population is based on existing body of research (references 20-32 and 42-44 in the manuscript) demonstrating that performance in behavioural tasks in the considered populations (controls and people at-risk of or experiencing psychosis) is significantly different and the differences have large effect sizes. The large effect sizes are further confirmed by the analysis of the collected data presented in the manuscript (column ‘Effect size’ in Table 5). Indeed, if the effect sizes of the differences between the cohorts in terms of the features used for classification were smaller, we would not achieve such excellent classification results.

Our aim of presenting the classification results, along the analysis of differences between cohorts, was to demonstrate that the large group difference (observed in our current study and known from literature) can be used to successfully discriminate between the two cohorts at the level of individuals.

Our original choice of the k-nearest neighbours classifier (kNN) was based on its simplicity and minimal number of hyperparameters. Namely, k – the number of the nearest neighbours considered, and the choice of distance metric used to compute distance between neighbours. kNN classifier used in the manuscript does not have any learned weights and treats all the features equally.

In the revised manuscript we compare 5 different types of classifiers. In addition to presented comparison of kNN and naive Bayes (NB) classifiers we also included support vector machine, bagged trees and linear discriminant (LD) classifiers. For the kNN and LD classifiers we, further, analysed how their performances depends on the number of features. We compared kNN with 3 and 18 features and LD with 2, 3 and 18 features. Results of the analysis demonstrated that the classification results are virtually independent from the choice of the model. In all the cases the classifiers have AUC in the range of 0.84-0.92 for the hold-out training-testing split and 0.84-0.91 for the leave-one-out training-testing split. We used two types of training-testing split to show the robustness of our results (training set size was n=48 for the hold-out training-testing split and n=63 for the leave-one-out training-testing split).

To avoid overfitting and to satisfy assumptions of some of the classifiers (e.g., NB assume that the features are independent) for all the classifiers we used only 3 out of 18 features (one from each task). Feature selection was data driven and based on the effect sizes of the differences between the cohorts.

Analysis of how performance of kNN and LD depends on the number of features revealed that:

1. performance of the kNN classifier (for which the features are collapsed into a single distance/similarity matrix) is practically indistinguishable for 3 and 18 features (with 18 features performing marginally better),
2. performance of the LD classifier is best for 3 features and decreased for 18 features and for 2 features (using only TMT and MG Leader-Follower data).

Worse performance of LD with 18 features can be a result of theoretical (‘curse of dimensionality’ [1]) or numerical (e.g., separating points in higher dimension might require estimation of more complex decision boundaries) limitations.

All presented classification results are obtained using default versions/ settings/ hyperparameters of the classifiers. The performance of all classifiers could potentially be improved by optimising their hyperparameters. As we are aware that the presented models (i.e., sets of coefficients of the trained classifiers) are not ready yet to be used in clinical applications (small sample size and study design) and to be conservative in our analysis we decided not to optimise the hyperparameters.

Classifiers for any future clinical applications should be fully optimised based on a larger sample and using a set of features extracted from a larger number of behavioural tasks paying careful attention not to overfit.

We also revised the manuscript for clarity and added references 50, 51, 52 and 65 for the added classification methods and additional analysis.

[1] Trunk GV. A problem of dimensionality: A simple example. IEEE Transactions on pattern analysis and machine intelligence. 1979 Jul(3):306-7.

In addition to the reviewers' comments, please revise the title of the manuscript to adequately reflect the study design and small sample size.

The new title of the paper is:

The potential of digital behavioural tests as a diagnostic aid for psychosis

Reviewer #1: The topic is relevant as psychotic illness is rising globally. The authors do a commendable job of trying innovative and cost-effective testing methods to timely identify the illness by the general practitioners and consequent referrals to treat it.

Major issues with this study stem from its generalizability, due to very small sample size of cohorts.

Generalisability of the study is ensured by the existence of well documented differences in performance in behavioural tasks between the cohorts included in the study (references 20-32 and 42-44 in the manuscript). Furthermore, in the current study we confirmed the presence of the large effect sizes (column ‘Effect size’ in Table 5). Please see also the reply to the comment by the Editor.

Also, the mean age is at the lower end, which means the samples may not have much representation of the older population.

We would like to note that the mean age of onset of psychosis is 22 years for men and 26 years for women (reference 1 in the manuscript). Mean ages of service users (28.8) and controls (24.2) cohorts are representative for the onset of psychosis and reflect clinical practice. The ages of the cohorts in our study might be slightly higher than reported in the literature because inclusion criteria specified that participants have to be at least 18 years old. Early [1] and late [2,3] onset psychoses are separately defined conditions that require different diagnostic approaches and are beyond the scope of the study.

[1] Downs, Johnny, et al. "Negative symptoms in early-onset psychosis and their association with antipsychotic treatment failure." *Schizophrenia bulletin* 45.1 (2019): 69-79. <https://doi.org/10.1093/schbul/sbx197>

[2] Leuchter, Andrew F., and James E. Spar. "The late-onset psychoses clinical and diagnostic features." *The Journal of nervous and mental disease* 173.8 (1985): 488-494. <https://doi.org/10.1097/00005053-198508000-00006>

[3] Brunelle, Sarah, Martin G. Cole, and Michel Elie. "Risk factors for the late‐onset psychoses: a systematic review of cohort studies." *International journal of geriatric psychiatry* 27.3 (2012): 240-252. <https://doi.org/10.1002/gps.2702>

Also, looks like participants with only English as the first language are selected in the studies.

This is due to characteristic of the region where the study took place and the patient population. In the case of the control population this was required to try to minimise the chances of misinterpretations due to the extensive use of questionnaires in the study.

We would like to add that the behavioural task themselves are language independent. This fact, if they would get widely adopted, would greatly facilitate comparison of research in different populations.

Recommend that authors provide the evidence of data sufficiency and generalizability of the results over the age, education level, and language.

We believe that age of our cohorts is representative for the onset of psychosis. The language limitation directly reflects our study design and is beyond our control. We addressed the level of education limitations in the section ‘Study limitations’ of the manuscript. The effect of the education level in the cohorts would need to be explored in future larger studies.

We would like to add that by openly sharing our methodology we hope to encourage replication studies in other parts of the world. We are also willing to actively support any researchers that would contact us with regards to replication of the study.

With the limited number of participants in the cohort, the spread over regions might be too thin, data is not available to conclude this.

The study took part in two NHS trusts in Bristol and Devon. In fact, most of the data is from Devon Partnership NHS trust (30 service users). We think that we have sufficient regional coverage of a single region in England.

Given above limitations, the pilot using the methods described is interesting, but need broader testing before putting into practice. Given the implications of this study on Home Health and care-coordination and care referral, authors are encouraged to enrich the implications section.

We agree with the reviewer that the presented approach requires further testing before putting in the practice. We are actively working on preparations of such studies. We agree with the reviewer that the study has wide-ranging potential implications for mental healthcare. We have now mention this in the implication section.

Reviewer #2: The authors proposed to use digital behavioural tests for early psychosis prediction. They extracted 18 features derived from the ‘mirror game’ (MG) and trail making task (TMT) and employed k-nearest neighbours (kNN) classifier for the prediction. After the inclusion and exclusion criteria the number of the positive and negative cases were 32 each. Therefore, they employed 25% hold-out and 1000 folds for the evaluation.

Strength:

1, The prediction problem was justified and well motivated.

2, The methodology was sound and the results looked reasonable.

Weakness:

1, The data size of 64 was a unfortunate limitation of this study. The generalization of the model could be doubtful.

Please see also responses to editor’s and reviewer #1 comments.

2, Only one model was presented. The authors could try a number more standard machine learning algorithms such as SVM.

In the revised manuscript we compared 5 different types of classifiers. In addition to presented comparison of kNN and naive Bayes classifiers we also included support vector machine, bagged trees and linear discriminant (LD) classifiers. For the kNN and LD classifiers we, further, analysed how their performance depends on the number of features. In all the cases the AUC is in the range of 0.84-0.92 for the hold-out training-testing split and 0.84-0.91 for the leave-one-out training-testing split, with the LD classifier having the highest AUC for the hold-out training-testing split. All presented classification results were obtained using default versions/ settings/ hyperparameters of the classifiers. Detailed comparison of the classifiers’ performance can be found in the updated Table 4. See also response to the Editor’s comments.

3, Model explainability was not explored. What were the key features that affected the model the most in its decision making?

Rather than analysing the features importance using one of many of available algorithms (e.g., minimum redundancy maximum relevance (MRMR), ranking based on chi-square tests, neighbourhood component analysis) we decide to analyse the effect sizes of the differences between the cohorts in terms of the features used for classification. The effect sizes are directly related to the differences (distance) between feature values (coordinates in the feature space) and affect performance of all the classifiers considered in the original and revised manuscript. Comparing effect sizes allowed us to not only quantify the importance of the individual features but also more generally of the three behavioural tasks considered in the manuscript. In the revised manuscript we add a paragraph in DISCUSSION that discusses importance of the features and classifiers’ interpretability in terms of effect sizes of differences between the features.

Suggestions:

1, The authors are encouraged to try a number of standard machine learning algorithms such as SVM.

Done. Please see response to weakness 2.

2, Explore model learned weights or other methods to improve model explainability.

Done. Please see response to weakness 3.

3, Any reporting of experiments or findings should be in past tense.

We revised tenses used in the manuscript.
