# Supplementary material for: The potential of digital behavioural tests as a diagnostic aid for psychosis
Source: PLOS Digit Health. 2023 Sep 15;2(9):e0000339. doi: 10.1371/journal.pdig.0000339 (PMC10503702; doi:10.1371/journal.pdig.0000339)
Supplement: Attachment — Submitted filename: response_2.docx [file pdig.0000339.s002.docx]

We would like to thank the reviewers for the comments that helped to clarify manuscript.

Reviewer #2: The authors have addressed most issues. Particularly, they added the Cliff's Delta effect size to demonstrate the strength of relationship between the dependent and independent variables, which is a sample size independent measure. I am not sure if it can explain away the issue of generalizability of machine learning models on a small data set.

The generalisability of our study results in terms of identifying a set of measures that could be used as features for reliable identification of patients at risk of developing psychosis is clearly supported (in a statistical sense) by the large effect sizes of the differences between the groups we consider. Furthermore, to show that our classification results reflect genuine differences observed in the data and do not depend on a specific ML approach (or a specific trained ML model) we demonstrated that multiple well established machine learning algorithms (with default settings) perform equally well. It is our future aim and aspiration to employ more tailored ML approaches trained on larger clinical samples and optimised paying careful attention not to overfit.

Reviewer #3: Small Sample Size: The study uses a small sample size (n=32 for both groups). This restricts the generalizability of the results and may not represent the heterogeneity of individuals with psychotic illnesses.

Although the group may not represent the full heterogeneity of individuals with psychotic illnesses or risk of developing psychosis, we believe it to be representative for the service users of specialist teams for early psychosis in Devon (a county in southwest England). The broad inclusion criterium of being accepted for an assessment for psychosis or risk of developing psychosis allowed us to recruit participant with a range of CAARMS (comprehensive assessment of at-risk mental state) scores (score=0, n=16; score=2, n=4; score=4, n=12).

The sample size of the service users (SU) group (n=32) was limited by the study design (its duration and geographical range). We recruited as many eligible SU as possible for the duration of the study (10 months of recruitment) in mental health NHS trusts in Bristol and Devon. The control group (n=32) was matching the SU by age and gender as closely as possible.

Overfitting Risk: The study doesn't mention cross-validation across multiple datasets, only a hold-out set. This could result in overfitting and may fail to generalize to unseen data.

We agree with the reviewer that with any ML classification task there is a risk of overfitting and failure of generalisation to unseen data.

Our study design restricts us to a single dataset. In such a case using a hold-out set (a set of data that is not being used to optimise the classifier in anyway) is considered a best practice. We believe that by successfully applying multiple unoptimised machine learning approaches and using two different types of hold-out sets (25% hold-out and leave-one-out), we minimise the risk of overfitting and demonstrated that the data supports the generalisability of the results rather than a specific ML model per se. In addition, we used 1000 folds to show the variability (uncertainty) of the results based on the 25% hold-out sets. Median values reported in Table 4 clearly show that the classification results are robust, while the 95% coverage interval illustrates the variability of the performance (uncertainty) of the classifiers for the unseen data (for a balanced training set n=48).

We hope our study will encourage collection of larger datasets in independent studies and locations in the future. These could then be used for developing a robust and interpretable ML model.

Limited Test Effectiveness: The mirror game and trail making task were found unsuitable in isolation to differentiate between critical subgroups (those at risk or with first episode psychosis). Given the variability in psychotic states, additional or different tests might be needed.

As stated in the abstract, results and discussion we agree with the reviewer. Any future clinical applications should be fully optimised based on a larger sample and using a set of features extracted from a larger number of behavioural tasks including best clinical practice and domain experts’ knowledge.

Feasibility Concerns: Implementation feasibility in a real-world setting isn't discussed. This includes patient comfort with technology, practitioner technological literacy, and the true cost of equipment and implementation.

As a part of the study, we collected feedback from the participants and practitioners regarding comfort with technology and technological literacy. 65% of participants found the test acceptable as a part of a routine clinical assessment for risk of psychosis (answer >5 on a 10-point scale to a question: ’How acceptable would you find the activity as a part of a routine clinical assessment for risk of psychosis?’) with only 5 out of 32 participants giving score <5. 87.5% of participants replied that they would be comfortable taking such a test at home (answer >5 on a 10-point scale to a question: ‘Would you be comfortable taking such a test at home?’) with 17 out of 32 participants giving a score 9 or 10 and only 2 participants giving score <5. Only clinical practitioners (doctors or research nurses) collected the data from the patients and all of them given positive oral feedback about the tasks and the data collection set-up. They particularly praised its portability (ability to run the tests at service users’ homes) and easy set-up (simply plugging in the USB cables into a computer).

We included two paragraphs in discussion describing the feasibility of real-world implementation in the revised manuscript. We also included prices of the equipment at the time of buying (prices at mid 2018, laptop 722.76 GBP, leap motion sensor 84.24 GBP). Health economic analysis of the true costs of real-world implementation is beyond the scope of current study.

Lack of Validation Against Clinical Diagnosis: It's not clear if the results were validated against a clinical diagnosis made by a psychiatrist. Without this, the applicability of results in a clinical setting is uncertain.

Yes, our results were validated against CAARMS, which is one of the gold standards in assessing service users at risk of or with first episode of psychosis. The study took place in a clinical setting (Devon Partnership NHS Trust (DPT) and Avon and Wiltshire Mental Health Partnership NHS Trust (AWP)), mental state of the service users was assessed at the CAARMS (comprehensive assessment of at-risk mental state) appointment with a consultant psychiatrist or a trained specialist with experience in at-risk mental states. CAARMS is one of NICE (National Institute for Health and Care Excellence) recommended psychosis risk assessment tool (https://www.england.nhs.uk/wp-content/uploads/2023/03/B1954-implementing-the-early-intervention-in-psychosis-access-and-waiting-time-standard.pdf). Our study design did not include follow-up contacts with participants.

The control group was not assessed by a psychiatrist. Control participants were screened using Patient Health Questionnaire-9 (PHQ-9) and Community Assessment of Psychic Experiences-42 (CAPE-42) questionnaires to ensure their suitability for inclusion in the study.

Privacy and Ethics: Digital tests often require collection of sensitive patient data, which raises privacy and ethical concerns not addressed in the study.

We envisage that most immediate potential future implementations of these test will be taking place within a healthcare setting (clinic, practitioners office). In such a case we suppose that the data collected during the test will be treated as any other patients’ data collected during medical examination. We envisage the tests as a decision support tool for clinicians and healthcare practitioners. We think that augmenting neuropsychological evaluation by means of digital batteries of behavioural test will be saving clinicians time for meaningful conversation with a help-seeking service user.

Indeed, any future deployment would require careful consideration of privacy and related ethical issues. We have now discussed the privacy and ethics concerns in the section describing the feasibility of real-world implementation.

Added paragraphs:

**Feasibility of the real-world implementation**

As a part of the study, we collected feedback from the participants and healthcare practitioners regarding acceptability and easiness of use of the tests. 65% of participants found the test acceptable as a part of a routine clinical assessment for risk of psychosis (answer >5 on a 10-point scale, 1 – not at all and 10 – very much, to a question: ’How acceptable would you find the activity as a part of a routine clinical assessment for risk of psychosis?’) with only 5 out of 32 participants giving a score <5. 87.5% of participants replied that they would be comfortable taking such a test at home (answer >5 on a 10-point scale to a question: ‘Would you be comfortable taking such a test at home?’) with 17 out of 32 participants giving a score 9 or 10 and only 2 participants giving a score <5 (all questions and SU answers are available at https://osf.io/rnzys/). The healthcare practitioners (doctors and research nurses) who collected the data from the service users provided oral feedback about the tasks and the data collection set-up, they praised its portability (ability to run the tests at service users’ homes) and easy set-up (simply plugging in the USB cables into a laptop computer).

We envisage that most immediate potential future implementations of digital batteries of behavioural tests will be taking place within a healthcare setting, in a clinic or practitioners office). We foresee the tests as part of a decision support tool and to be employed as any other medical test. The data collected during the test will be treated as any other patients’ data collected during medical examination in terms of ethics and privacy. We believe that augmenting neuropsychological evaluation by means of digital batteries of behavioural test could be saving clinicians time for meaningful conversation with a help-seeking service user. We hope that the time saved by the tests, together with their demonstrated accuracy will help to humanise and destigmatise mental health diagnosis. We are aware that future deployment of such technology would require careful consideration of privacy and related ethical issues, which is beyond the scope of this paper.
